# Association of liver function markers and apolipoprotein E ε4 with pathogenesis and cognitive decline in Alzheimer’s disease

**DOI:** 10.3389/fnagi.2024.1411466

**Published:** 2024-07-24

**Authors:** Sang-Won Han, Sang-Hwa Lee, Jong Ho Kim, Jae-Jun Lee, Young Ho Park, SangYun Kim, Kwangsik Nho, Jong-Hee Sohn

**Affiliations:** ^1^Department of Neurology, Chuncheon Sacred Heart Hospital, Hallym University College of Medicine, Chuncheon-si, Gangwon-do, Republic of Korea; ^2^Department of Anesthesiology and Pain Medicine, Chuncheon Sacred Heart Hospital, Hallym University College of Medicine, Chuncheon-si, Gangwon-do, Republic of Korea; ^3^Institute of New Frontier Research Team, Hallym University, Chuncheon-si, Gangwon-do, Republic of Korea; ^4^Department of Neurology, Seoul National University College of Medicine and Seoul National University Bundang Hospital, Seongnam-si, Gyeonggi-do, Republic of Korea; ^5^Department of Radiology and Imaging Sciences, Center for Computational Biology and Bioinformatics, Indiana Alzheimer’s Disease Research Center, Indiana University School of Medicine, Indianapolis, IN, United States

**Keywords:** Alzheimer’s disease, amyloid-β, *APOE* ε4, cognition, liver enzymes

## Abstract

**Background:**

Alzheimer’s disease (AD) is a complex neurodegenerative disorder influenced by various factors, including liver function, which may impact the clearance of amyloid-β (Aβ) in the brain. This study aimed to explore how the apolipoprotein E (*APOE*) ε4 allele affects the relationship of liver function markers with AD pathology and cognition.

**Methods:**

We analyzed data from two independent cohorts, including 732 participants from the Hallym University Medical Center and 483 from the Alzheimer’s Disease Neuroimaging Initiative, each group consisting of individuals with and without the *APOE* ε4 allele. Cross-sectional analyses evaluated the associations of liver enzymes (aspartate aminotransferase [AST], alanine aminotransferase [ALT], alkaline phosphatase, total bilirubin, and albumin) with AD diagnosis, amyloid positron emission tomography (PET) burden, and cerebrospinal fluid biomarkers for AD (Aβ42, total tau, and phosphorylated tau181) at baseline. Longitudinally, we investigated the associations between these liver enzymes and changes in cognitive performance over the course of a year. Logistic and linear regression models were used to analyze these associations and mediation analyses were conducted to assess whether age and amyloid PET burden mediated these associations.

**Results:**

Only in the *APOE* ε4 carrier group, a high AST to ALT ratio and low ALT levels were significantly associated with AD diagnosis, increased amyloid PET burden, and faster longitudinal decline in cognitive function in both cohorts. In particular, the AST to ALT ratio was associated with cerebrospinal fluid Aβ42 levels exclusively in the *APOE* ε4 carrier group in the Alzheimer’s Disease Neuroimaging Initiative cohort but not with phosphorylated tau_181_ or total tau levels. Moreover, mediation analyses from both cohorts revealed that in the *APOE* ε4 carriers group, age did not mediate the associations between liver enzymes and AD diagnosis or amyloid PET burden. However, amyloid PET burden partially mediated the association between liver enzymes and AD diagnosis exclusively in the *APOE* ε4 carriers group.

**Conclusion:**

This study provides valuable insights into the significant association of the *APOE* ε4 allele with liver enzymes and their potential role in Aβ-related pathogenesis and cognition in AD. Further research is required to elucidate the underlying mechanisms and potential therapeutic implications of these findings.

## 1 Introduction

Alzheimer’s Disease (AD) is the most common form of dementia, characterized by progressive neurodegeneration leading to cognitive decline (Long and Holtzman, 2019). AD is characterized by neuropathological markers, including accumulation of extracellular amyloid-β (Aβ) in the form of neuritic plaques and intracellular deposition of hyperphosphorylated tau in neurofibrillary tangles (Long and Holtzman, 2019). AD is a complex neurodegenerative disorder due to its multifactorial etiology, involving a combination of genetic susceptibilities and environmental factors (Hampel et al., 2010).

Despite advances in understanding the molecular pathogenesis of AD, current diagnostic approaches and therapeutic interventions remain inadequate. Recent investigations have focused on the potential interactions between the brain and peripheral organs, particularly the liver, in relation to Aβ clearance (Estrada et al., 2019; Bassendine et al., 2020; Cheng et al., 2020; Huang et al., 2022). This attention to peripheral organ involvement unveils novel avenues for understanding pathophysiology of AD and identifying accessible biomarkers. In particular, liver function enzymes, such as aspartate aminotransferase (AST), alanine aminotransferase (ALT), and alkaline phosphatase (ALP), have been explored for their association with AD, cognitive function, and Aβ accumulation (Kellett et al., 2011; Nho et al., 2019; Lu et al., 2021; Ferri et al., 2022; Han et al., 2022; Li et al., 2022). However, conflicting findings from other studies have challenged this association (Kamada et al., 2016; Vasantharekha et al., 2017). These inconsistencies could be attributed to differences in the study designs, sample sizes, population demographics, methodologies, the specific liver function markers assessed, and the statistical approaches employed. Importantly, previous studies did not account for genetic factors, such as the apolipoprotein E (*APOE*) genotype, which significantly influences AD pathogenesis. These findings suggest a complex relationship between liver function and AD pathogenesis, prompting the need for more exploration.

Among the genetic factors implicated in AD, the *APOE* ε4 allele has been identified as the most significant genetic risk factor, increasing disease risk in a gene dose-dependent manner (Farrer et al., 1997). The *APOE* gene encodes APOE, a glycoprotein with crucial roles in lipid transport and metabolism, both in the brain and periphery (Liu et al., 2013; Chernick et al., 2019; Muñoz et al., 2019). Notably, APOE4 has been shown to inhibit peripheral Aβ clearance and increase Aβ accumulation in the brain, a hallmark of AD pathology (Sharman et al., 2010; Liu et al., 2022). However, the interplay between *APOE* ε4 status, liver enzyme levels, and AD, particularly in the context of Aβ accumulation and cognitive functions, remains poorly understood. Furthermore, the potential mediation effects of age and Aβ burden on these associations, contingent upon *APOE* ε4 carrier status, have not been adequately addressed. These considerations are crucial, given the liver function in systemic metabolism and its potential influence on AD pathogenesis.

Our study aimed to address this knowledge gap by investigating the association of *APOE* ε4 carrier status and blood liver enzymes, including AST, ALT, the AST to ALT ratio, ALP, total bilirubin, and albumin, with AD diagnosis, AD biomarkers, and cognitive performance, in two independent cohorts. Additionally, if such associations were observed, we investigated whether these relationships were independent or mediated by age or Aβ burden.

## 2 Methods

### 2.1 Study participants

Two independent cohorts were used in this study: the Hallym University Medical Centers (HUMC) cohort and the Alzheimer’s Disease Neuroimaging Initiative (ADNI) cohort. In the HUMC cohort, we conducted a retrospective analysis of clinical data using the Smart Clinical Data Warehouse, a comprehensive big-data analytical solution specifically designed for clinical applications. This system collects data from four medical university-affiliated hospitals located in various provinces of the Republic of Korea (Hallym Sacred Heart Hospital, Dongtan Sacred Heart Hospital, Kangnam Sacred Heart Hospital, and Chuncheon Sacred Heart Hospital). The Smart Clinical Data Warehouse, underpinned by the QlikView Elite Solution (Qlik, King of Prussia, PA, USA), facilitates in-depth analysis of electronic medical record text data and integrated analysis of static data. The selection of participants for the HUMC cohort was based on clinical visits for symptoms of cognitive decline at one of four associated hospitals under HUMC during the period from November 2015 to June 2023. All participants underwent comprehensive assessments, including physical and neurological examinations, laboratory tests, APOE genotyping, neuropsychological assessments, brain magnetic resonance imaging, and amyloid positron emission tomography (PET) imaging to provide a robust dataset for analysis. We excluded individuals who had not undergone liver function tests within 1 year of their amyloid PET imaging date as well as those with concomitant medical conditions, such as hepatocellular carcinoma and liver cirrhosis (which could significantly affect liver function) or other neurodegenerative disorders, including frontotemporal dementia (Gorno-Tempini et al., 2011; Rascovsky et al., 2011), corticobasal syndrome (Armstrong et al., 2013), Parkinson’s disease dementia (Dubois et al., 2007), and progressive nuclear palsy (Litvan et al., 1996). Additionally, we excluded participants who had outlier values in liver function parameters, including AST, ALT, ALP, total bilirubin, and albumin which exceeded the interquartile range by four times, due to the potential for significant liver function impairment. Following these exclusions, the AST to ALT ratios were calculated from the refined dataset. The study protocol was approved by the Clinical Research Ethics Committee of Chuncheon Sacred Heart Hospital and Hallym University and conformed to the principles outlined in the Declaration of Helsinki.

In the ADNI cohort, the initial phase (ADNI-1) (Petersen et al., 2010) was launched in 2003 by the National Institute on Aging, National Institute of Biomedical Imaging and Bioengineering, Food and Drug Administration, private pharmaceutical companies, and nonprofit organizations. The primary objective was to investigate the feasibility of using serial magnetic resonance imaging, PET, other biological markers, and clinical and neuropsychological assessments as dependable *in vivo* indicators of AD pathogenesis. Subsequent phases, namely ADNI-GO (ADNIGO, 2009; Aisen et al., 2010), ADNI-2 (Aisen et al., 2015), and ADNI-3 (Weiner et al., 2017), extended the initial phase, allowing for the ongoing follow-up of existing participants and inclusion of new enrollments. A comprehensive description of ADNI, up-to-date information, inclusion and exclusion criteria, clinical and neuroimaging protocols, and a summary of the diagnostic criteria are available at https://www.adni-info.org. Demographic and clinical information, raw neuroimaging data, cerebrospinal fluid (CSF) biomarker data, information on *APOE* ε4 carrier status, and cognitive scores were obtained from the ADNI Laboratory of Neuro Imaging (LONI) website^1^ (Saykin et al., 2015). Written informed consent was obtained from all participants at enrollment, including consent for data analysis and sharing. This study was approved by the Institutional Review Board of each participating site. We specifically selected participants underwent both serum liver function tests and APOE genotyping. Furthermore, we excluded participants with outlier values in liver function parameters, including AST, ALT, ALP, total bilirubin, and albumin which exceeded the interquartile range by four times. Subsequent to these exclusions, we calculated the AST to ALT ratios from the refined dataset.

In both the HUMC and ADNI cohorts, all participants underwent neuropsychological assessments annually to provide a longitudinal perspective on changes in cognitive function over time. In contrast, measurements of liver enzymes, CSF biomarkers for AD, and amyloid PET imaging were performed only at baseline.

### 2.2 Diagnostic status

In the HUMC cohort, participants were categorized as having probable AD based on the National Institute on Aging–Alzheimer’s Association criteria (McKhann et al., 2011), mild cognitive impairment (MCI) according to the National Institute on Aging–Alzheimer’s Association criteria (Albert et al., 2011), or subjective cognitive decline (SCD) in accordance with the guidelines of Jessen et al. (2014).

Participants in the ADNI cohort who met the criteria for a clinical diagnosis of probable AD, MCI, and cognitively normal (CN) older individuals were prospectively followed up with clinical data, neuroimaging studies, and biological samples gathered for molecular biomarker measurements, as previously described (ADNIGO, 2009; ADNI1, 2010; Weiner et al., 2017; ADNI2, 2020). Briefly, the Logical Memory from the Wechsler Memory Scale—Revised (Wechsler, 1987), Mini-Mental State Examination (MMSE) (Folstein et al., 1975), and Clinical Dementia Rating scale (Morris, 1993) were used to determine the diagnostic classifications.

### 2.3 Amyloid PET imaging

In the HUMC cohort, the amyloid status was determined using amyloid PET imaging with [18F] florbetaben (*n* = 561) and [18F] flutemetamol (*n* = 171) tracers. The amyloid PET status was categorized as positive (abnormal) or negative (normal) based on visual ratings by one nuclear medicine physician and one neurologist, both experienced and trained in the field. [18F] florbetaben PET images were classified as positive based on a visual assessment scoring of 2 or 3 according to the brain Aβ plaque load (BAPL) scoring system (Barthel et al., 2011). For [18F] flutemetamol PET images, visual interpretation involved a systematic review across five brain regions: frontal, parietal, posterior cingulate and precuneus, striatum, and lateral temporal areas. A scan was considered positive if any of these regions showed Aβ deposition in either hemisphere (Farrar et al., 2019). The concordance rate between CSF Aβ42 and BAPL scores was 77.4%, using an Aβ42 cutoff of less than 600 pg/mL (Spallazzi et al., 2019). The raters were unaware of the clinical details of the participants but had knowledge of the specific PET tracer used for each image. In case of discordance, the raters held discussions to reach a consensus.

In the ADNI cohort, preprocessed [18F] florbetapir PET scans were obtained from the ADNI LONI site (see text footnote 1), following previously reported methods of PET scan acquisition and processing (Jagust et al., 2010, 2015). [18F] Florbetapir PET standard uptake value ratio (SUVR) values were used to evaluate amyloid deposition by normalizing the intensity using the whole cerebellar reference region.

### 2.4 Liver function enzymes

In the HUMC cohort, all participants underwent venous blood sample collection following a 12-h overnight fast. These samples were obtained to assess serum levels of liver function enzymes, including AST [normal range, <40 U/L (Neuschwander-Tetri et al., 2008)], ALT [normal range, <40 U/L (Neuschwander-Tetri et al., 2008)], total bilirubin [normal range, 0.2–1.0 mg/dL (Franchini et al., 2010)], ALP [normal range, 30–115 U/L (Lum, 1995)], and albumin [normal range, 3.5–5.0 g/dL (Doweiko and Nompleggi, 1991)]. An AST/ALT ratio of greater than two suggests cirrhosis in various liver diseases (Nyblom et al., 2006). The samples were analyzed using an automated blood analyzer (Beckman Coulter AU5800, Beckman Coulter Inc., Brea, CA, USA), which was operated within the Department of Laboratory Medicine at each of the participating hospitals (Hallym Sacred Heart Hospital, Dongtan Sacred Heart Hospital, Kangnam Sacred Heart Hospital, and Chuncheon Sacred Heart Hospital).

In the ADNI cohort, blood samples were obtained from the participants during fasting and handled according to the established laboratory standard operating procedures of the ADNI (Kang et al., 2015). Results of liver enzymes, including AST, ALT, ALP, total bilirubin, and albumin, were acquired from the ADNI data repository and subsequently incorporated into this study.

### 2.5 CSF biomarkers

In the ADNI cohort, CSF samples were obtained through lumbar puncture conducted in the morning following an overnight fast. The collected samples were frozen within 1 h of collection and transported on dry ice to the ADNI Biomarker Core Laboratory at the University of Pennsylvania Medical Center. In the laboratory, CSF samples were measured in pristine aliquots using the multiplex xMAP Luminex platform (Luminex Corp, Austin, TX) (Olsson et al., 2005) with immunoassay kit–based reagents containing monoclonal antibodies targeting Aβ42, total tau (t-tau), and phosphorylated tau_181_ (p-tau_181_), as previously described (Shaw et al., 2009; Hansson et al., 2018). CSF biomarker data were downloaded from the ADNI LONI website, which is accessible at http://adni.loni.usc.edu. CSF biomarker data were log-transformed to reduce skewness.

### 2.6 Comprehensive neuropsychological assessment

In the HUMC cohort, a comprehensive series of neuropsychological assessments were performed longitudinally to evaluate individuals across two distinct cognitive domains: language and memory functions. The raw scores for each participant based on their performance on individual cognitive tests were converted into standardized z-scores, which were adjusted for age, sex, and education norms (Kang et al., 2003). For each participant, based on their performance on individual cognitive tests, the z-scores for language and memory functions were calculated. Specifically, language function was assessed using the Korean Boston Naming Test (Kim and Na, 1999) and memory function was evaluated using the Seoul Verbal Learning Test 20-min delayed recall test.

In the ADNI cohort, composite scores were used to assess language and memory functions. For the language assessment, a composite score was calculated using the Boston Naming Test (Kaplan et al., 2001), animal and vegetable fluency, language components of the MMSE (Folstein et al., 1975), memory tasks of the Alzheimer’s Disease Assessment Schedule–Cognition (ADAS-Cog) (Mohs et al., 1997), and phonemic fluency and sentence repetition of the Montreal Cognitive Assessment (Nasreddine et al., 2005). The memory function was assessed using a composite score that incorporated memory-related tasks from the ADAS-Cog (Mohs et al., 1997), the Rey Auditory Verbal Learning Test (Rey, 1958), memory components of the MMSE (Folstein et al., 1975), and the Logical Memory task (Wechsler, 1987). These composite scores were standardized to have a mean value of 0 and a standard deviation of 1.20, allowing for a consistent and comparative assessment of cognitive function across different individuals in the ADNI cohorts.

### 2.7 Statistical analysis

We performed a comprehensive analysis to compare the baseline characteristics of individuals in both the amyloid-negative and amyloid-positive groups as well as in the *APOE* ε4 carrier and *APOE* ε4 non-carrier groups. We used chi-squared tests to examine categorical variables and Mann–Whitney U tests or *t*-test to assess continuous variables, as appropriate.

We conducted a logistic regression analysis to investigate the association of each liver enzyme with AD diagnosis and amyloid PET status. To analyze the association between liver enzymes and longitudinal changes in cognitive performance, we used linear mixed-effects models (Pinheiro and Bates, 2006). We conducted linear regression analyses to investigate the association of liver enzymes with amyloid PET global SUVR and CSF biomarkers for AD, including Aβ42, p-tau_181_, and t-tau.

In accordance with established methodologies (Hayes, 2009), we performed mediation analyses of liver function markers demonstrating a significant correlation with AD diagnosis and amyloid PET burden to investigate whether the identified association was potentially mediated by age. Additionally, we conducted mediation analyses focusing on liver function markers revealing a significant correlation with AD diagnosis to investigate whether the amyloid PET burden potentially mediated this association. To conduct these analyses, we used the Mediation R package, which allowed us to evaluate indirect effects through a bootstrapping approach involving the generation of 10,000 non-parametric simulations (Tingley et al., 2014).

In the HUMC cohort, covariates for association analysis with AD diagnosis, amyloid PET status, and CSF biomarkers for AD as well as for mediation analysis included age, sex, hypertension, diabetes mellitus, dyslipidemia, and statin use for both *APOE* ε4 carrier and *APOE* ε4 non-carrier groups. Similarly, in the ADNI cohort, we included covariates such as age, sex, and body mass index (BMI) for both *APOE* ε4 carrier and *APOE* ε4 non-carrier groups. For the association analysis with cognitive performance, education was included along with the previously mentioned covariates.

All statistical analyses were performed using R version 4.2.0 (The R Foundation for Statistical Computing, Vienna, Austria). Statistical significance was defined as *p* < 0.05, with adjustments for multiple comparisons. For multiple testing adjustments, we used the Benjamini–Hochberg procedure for false discovery rate correction (Benjamini and Hochberg, 1995).

## 3 Results

### 3.1 Demographics and clinical characteristics

In the HUMC cohort, a total of 770 participants were initially included. Exclusions were made for 28 participants with outlier values in liver function parameters (AST above 63, ALT above 62, ALP above 189, total bilirubin above 1.7, and albumin below 2.8), four with hepatocellular carcinoma, and six with liver cirrhosis. Subsequently, the remaining participants were categorized into the *APOE* ε4 carrier (*N* = 218 [29.8%]) and *APOE* ε4 non-carrier (*N* = 514) groups. The *APOE* ε4 carrier group comprised 26 patients with SCD, 87 with MCI, and 105 with probable AD. The median age of the participants in this group was 74 years, and 70.6% were females. The *APOE* ε4 non-carrier group comprised 45 patients with SCD, 282 with MCI, and 187 with probable AD. The median age of the participants in this group was 74 years, and 66.1% were females. There were no significant differences in age, sex, and frequency of hypertension, diabetes mellitus, dyslipidemia, or statin use between the two groups.

In the ADNI cohort, a total of 498 participants were initially included. After exclusion of 15 participants with outlier values in liver function parameters (AST above 52, ALT above 63, ALP above 145, total bilirubin above 1.5, and albumin below 3.8), the cohort was divided into the *APOE* ε4 carrier (*N* = 200 [41.4%]) and *APOE* ε4 non-carrier (*N* = 283) groups. The *APOE* ε4 carrier group included 95 individuals with CN, 66 with MCI, and 39 with probable AD. The median age of the participants in this group was 70 years, and 55.0% were females. The *APOE* ε4 non-carrier group included 183 individuals with CN, 82 with MCI, and 18 with probable AD. The median age of the participants in this group was 70 years, and 54.4% were females. There were no significant differences in age, sex, or education level between the two groups. However, the *APOE* ε4 carrier group showed poorer performance in memory and language functions compared to the *APOE* ε4 non-carrier group in the HUMC cohort. Similarly, the *APOE* ε4 carrier group in the ADNI cohort exhibited lower performance in memory function than the *APOE* ε4 non-carrier group. Regarding detailed baseline characteristics, Tables 1, 2 present a comparison of participants in the HUMC and ADNI cohorts, respectively.

**Table 1:** Demographic and clinical characteristics, and laboratory results of participants in the HUMC cohort.

	*APOE* ε4 − (*n* = 514)			*APOE* ε4 ± (*n* = 218)			*APOE* ε4 ± (*n* = 732)			
	Amyloid PET − (*n* = 367)	Amyloid PET + (*n* = 147)	*P* ^a^	Amyloid PET − (*n* = 77)	Amyloid PET + (*n* = 141)	*P* ^a^	Amyloid PET − (*n* = 444)	Amyloid PET + (*n* = 288)	*P* ^a^	*P* ^b^
Female	241 (65.7)	99 (67.3)	0.716	53 (68.8)	101 (71.6)	0.664	294 (66.2)	200 (69.4)	0.362	0.235
Age, years	74.0 (66.5–80.0)	74.0 (67.0–80.0)	0.933	70.0 (66.0–78.0)	74.0 (68.0–78.0)	0.163	74.0 (66.0–80.0)	74.0 (67.0–79.0)	0.464	0.890
Education, years	9.0 (6.0–12.0)	9.0 (6.0–12.0)	0.061	9.0 (6.0–12.0)	9.0 (6.0–12.0)	0.254	9.0 (6.0–12.0)	9.0 (6.0–12.0)	0.048	0.045
Hypertension	87 (23.7)	35 (23.8)	0.980	16 (20.8)	26 (18.4)	0.676	103 (23.2)	61 (21.2)	0.522	0.185
Diabetes mellitus	62 (16.9)	18 (12.2)	0.189	15 (19.5)	20 (14.2)	0.309	77 (17.3)	38 (13.2)	0.132	0.868
Dyslipidemia	54 (14.7)	19 (12.9)	0.600	12 (15.6)	22 (15.6)	0.997	66 (14.9)	41 (14.2)	0.814	0.625
Statin use	72 (19.6)	28 (19.0)	0.883	21 (27.3)	32 (22.7)	0.451	93 (20.9)	60 (20.8)	0.971	0.140
Diagnosis SCD/MCI/AD	40/221/106	5/61/81		20/51/6	6/36/99		60/272/112	11/97/180		
AST, U/L	21.0 (18.0–26.0)	22.0 (19.0–26.5)	0.262	22.0 (18.0–25.0)	22.0 (19.0–27.0)	0.328	22.0 (18.0–26.0)	22.0 (19.0–27.0)	0.399	0.127
ALT, U/L	17.0 (12.0–22.0)	15.0 (12.0–20.5)	0.124	17.0 (14.0–22.0)	15.0 (13.0–19.0)	0.010	17.0 (12.0–22.0)	15.0 (12.0–20.0)	0.004	0.009
AST to ALT ratio > 2	1.30 (1.04–1.59) 28 (7.6)	1.48 (1.20–1.76) 14 (9.5)	<0.001	1.27 (1.07–1.43) 1 (1.3)	1.46 (1.27–1.67) 7 (5.0)	<0.001	1.29 (1.04–1.56) 29 (6.5)	1.47 (1.25–1.73) 21 (7.3)	0.001	<0.001
ALP, U/L	73.0 (59.5–87.0)	72.0 (59.0–87.5)	0.648	70.0 (57.0–82.0)	68.0 (58.0–83.0)	0.479	72.0 (59.0–87.0)	70.0 (58.0–85.0)	0.070	0.121
TB, mg/dL	0.50 (0.40–0.70)	0.50 (0.39–0.63)	0.880	0.50 (0.40–0.64)	0.50 (0.40–0.60)	0.972	0.50 (0.40–0.70)	0.50 (0.40–0.61)	0.742	0.778
Albumin, g/dL	4.40 (4.20–4.60)	4.40 (4.10–4.60)	0.146	4.40 (4.10–4.60)	4.40 (4.20–4.60)	0.850	4.40 (4.20–4.60)	4.40 (4.20–4.60)	0.485	0.371
Memory domain, Z scores	−1.21 (−1.86 – −0.34)	−2.08 (−2.73 – −1.64)	<0.001	−1.64 (−2.29 – −0.76)	−2.09 (−2.73 – −1.85)	<0.001	−1.41 (−1.87 – −0.34)	−2.09 (−2.73 – −1.65)	<0.001	<0.001
Language domain, Z scores	−0.42 (−1.39–0.42)	−0.99 (−1.97 – −0.07)	<0.001	−0.57 (−2.00–0.26)	−0.74 (−1.91–0.06)	0.388	−0.46 (−1.52–0.38)	−0.90 (−1.94–0.01)	<0.001	<0.001

Values are presented as median (interquartile range), mean ± standard deviation, or number (%), unless indicated otherwise.

^a^The Mann–Whitney U test or *t*-test was used to determine the *p* value for comparisons of continuous variables between amyloid negative and amyloid positive groups, as appropriate. For categorical variables, the chi-square test was used to determine the *p* value for comparisons between these two groups.

^b^The Mann–Whitney U test or chi-square test was used to determine the *p* value for comparisons between *APOE* ε4 negative and *APOE* ε4 positive groups, as appropriate.

AD, Alzheimer’s disease; ALT, alanine aminotransferase; ALP, alkaline phosphatase; APOE, apolipoprotein E; AST, aspartate aminotransferase; HUMC, Hallym University Medical Centers; MCI, mild cognitive impairment; PET, positron emission tomography; SCD, subjective cognitive decline; TB, total bilirubin.

**Table 2:** Demographic and clinical characteristics, and laboratory results of participants in the ADNI cohort.

	*APOE* ε4− (*n* = 283)	*APOE* ε4+ (*n* = 200)	*APOE* ε4± (*n* = 483)	*P* ^a^
Female	154 (54.4)	110 (55.0)	264 (54.7)	0.899
Age, years	71.2 ± 7.43	69.9 ± 6.93	70.7 ± 7.25	0.052
Education, years	16.0 (15.0–18.0)	16.0 (15.0–18.0)	16.0 (15.0–18.0)	0.485
BMI, kg/m^2^	26.8 (24.1–30.2)	25.8 (23.3–29.2)	26.3 (23.9–29.9)	0.017
Diagnosis CN/MCI/AD	183/82/18	95/66/39	278/148/57	
Amyloid PET global SUVR	1.01 (0.97–1.08)	1.24 (1.04–1.50)	1.05 (0.98–1.30)	<0.001
AST, U/L	25.0 (20.5–28.0)	25.0 (22.0–29.0)	25.0 (21.0–29.0)	0.156
ALT, U/L	23.0 (18.0–29.0)	24.0 (19.0–30.0)	23.0 (19.0–29.0)	0.114
AST to ALT ratio > 2	1.04 (0.87–1.25) 1 (0.4)	1.04 (0.85–1.25) 2 (1.0)	1.04 (0.86–1.25) 3 (0.6)	0.841
ALP, U/L	71.0 (59.0–86.0)	70.0 (58.0–84.0)	71.0 (59.0–85.0)	0.443
TB, mg/dL	0.40 (0.30–0.60)	0.40 (0.30–0.60)	0.40 (0.30–0.60)	0.518
Albumin, g/dL	4.50 (4.30–4.60)	4.50 (4.30–4.70)	4.50 (4.30–4.70)	0.058
CSF Aβ42, pg/mL	1,285 (868–1875)	747 (512–1,062)	1,034 (688–1,585)	<0.001
CSF p-tau, pg/mL	18.0 (14.1–24.1)	24.5 (18.0–31.8)	20.3 (15.0–28.1)	<0.001
CSF total tau, pg/mL	211 (170–277)	265 (198–341)	230 (178–305)	<0.001
Memory domain, Z scores	0.92 (0.26–1.59)	0.48 (−0.43–1.26)	0.80 (0.01–1.46)	<0.001
Language domain, Z scores	0.69 ± 0.57	0.65 ± 0.67	0.67 ± 0.61	0.515

Values are presented as median (interquartile range), mean ± standard deviation, or number (%), unless indicated otherwise.

^a^Mann–Whitney U test or *t*-test was used to determine the *p* value for comparisons of continuous variables between *APOE* ε4 negative and *APOE* ε4 positive groups, as appropriate. For categorical variables, the chi-square test was used to determine the *p* value for comparisons between these two groups.

Aβ, amyloid-β; AD, Alzheimer’s disease; ADNI, Alzheimer’s Disease Neuroimaging Initiative; ALT, alanine aminotransferase; ALP, alkaline phosphatase; APOE, apolipoprotein E; AST, aspartate aminotransferase; BMI, body mass index; CN, cognitively normal; CSF, cerebrospinal fluid; MCI, mild cognitive impairment; p-tau, phosphorylated tau; PET, positron emission tomography; SUVR, standard uptake value ratio; TB, total bilirubin.

### 3.2 Association analysis of liver enzymes with amyloid PET burden and diagnostic status

In the HUMC cohort, ALT levels (estimate for logistic regression coefficients = −0.076, standard error (SE) = 0.027, *p* = 0.01) were significantly lower in the amyloid-positive group (median value: ALT, 15 U/L; interquartile range (IQR): ALT, 13–19 U/L) compared to the amyloid-negative group (median value: ALT, 17 U/L; IQR: ALT, 14–22 U/L) in the *APOE* ε4 carrier group after adjustment for multiple comparisons (Table 3). In addition, the AST to ALT ratio (estimate = 2.412, SE = 0.574, *p* < 0.001) was significantly higher in the amyloid-positive group (median value: AST to ALT ratio, 1.46; IQR: AST to ALT ratio, 1.27–1.67) compared to the amyloid-negative group (median value: AST to ALT ratio, 1.27; IQR: AST to ALT ratio, 1.07–1.43) within the *APOE* ε4 carrier group. Regarding diagnostic status, ALT levels (estimate = −0.195, SE = 0.048, *p* < 0.001) were significantly decreased, while the AST to ALT ratio (estimate = 5.506, SE = 1.236, *p* < 0.001) was significantly increased in AD compared with SCD in *APOE* ε4 carrier group (Table 4). However, the *APOE* ε4 non-carrier group did not demonstrate significant associations between any of the six liver function markers and the diagnostic status or amyloid PET positivity.

**Table 3:** Results of the association analysis of liver function markers with amyloid PET positivity in the HUMC cohort.

	Amyloid PET positivity			
	*APOE* ε4−^a^		*APOE* ε4+^a^	
	Estimate^b^ (SE)	*P* ^c^	Estimate^b^ (SE)	*P* ^c^
AST, U/L	0.008 (0.013)	0.72	0.014 (0.024)	0.69
ALT, U/L	−0.015 (0.012)	0.44	−0.076 (0.027)	0.01
AST to ALT ratio	0.271 (0.181)	0.42	2.412 (0.574)	<0.001
ALP, U/L	−0.002 (0.004)	0.72	−0.010 (0.007)	0.34
TB, mg/dL	−0.086 (0.415)	0.84	0.097 (0.609)	0.87
Albumin, g/dL	−0.441 (0.298)	0.42	0.508 (0.454)	0.40

^a^After adjusting for age, sex, hypertension, diabetes mellitus, dyslipidemia, and statin use.

^b^Logistic regression coefficients of association between liver function markers and amyloid PET positivity.

^c^Adjusted *p* value for FDR to correct for 6 tests of associations between liver enzymes and amyloid PET positivity.

ALP, alkaline phosphatase; ALT, alanine aminotransferase; APOE, apolipoprotein E; AST, aspartate aminotransferase; FDR, false discovery rate; HUMC, Hallym University Medical Centers; PET, positron emission tomography; SE, standard error; TB, total bilirubin.

**Table 4:** Results of the association analysis of liver function markers with AD diagnosis in the HUMC cohort.

	Diagnostic status (SCD or AD)			
	*APOE* ε4−^a^		*APOE* ε4+^a^	
	Estimate^b^ (SE)	*P* ^c^	Estimate^b^ (SE)	*P* ^c^
AST, U/L	−0.026 (0.021)	0.37	0.016 (0.038)	0.82
ALT, U/L	−0.021 (0.018)	0.37	−0.195 (0.048)	<0.001
AST to ALT ratio	−0.150 (0.395)	0.82	5.506 (1.236)	<0.001
ALP, U/L	0.018 (0.009)	0.19	−0.001 (0.013)	0.94
TB, mg/dL	0.168 (0.752)	0.82	0.500 (1.095)	0.82
Albumin, g/dL	−1.118 (0.583)	0.19	0.527 (0.708)	0.82

^a^After adjusting for age, sex, hypertension, diabetes mellitus, dyslipidemia, and statin use.

^b^Logistic regression coefficients of association between liver function markers and diagnosis.

^c^Adjusted p value for FDR to correct for 6 tests of associations between liver enzymes and diagnosis.

AD, Alzheimer’s disease; ALP, alkaline phosphatase; ALT, alanine aminotransferase; APOE, apolipoprotein E; AST, aspartate aminotransferase; FDR, false discovery rate; HUMC, Hallym University Medical Centers; PET, positron emission tomography; SCD, subjective cognitive decline; SE, standard error; TB, total bilirubin.

**Figure 1:**
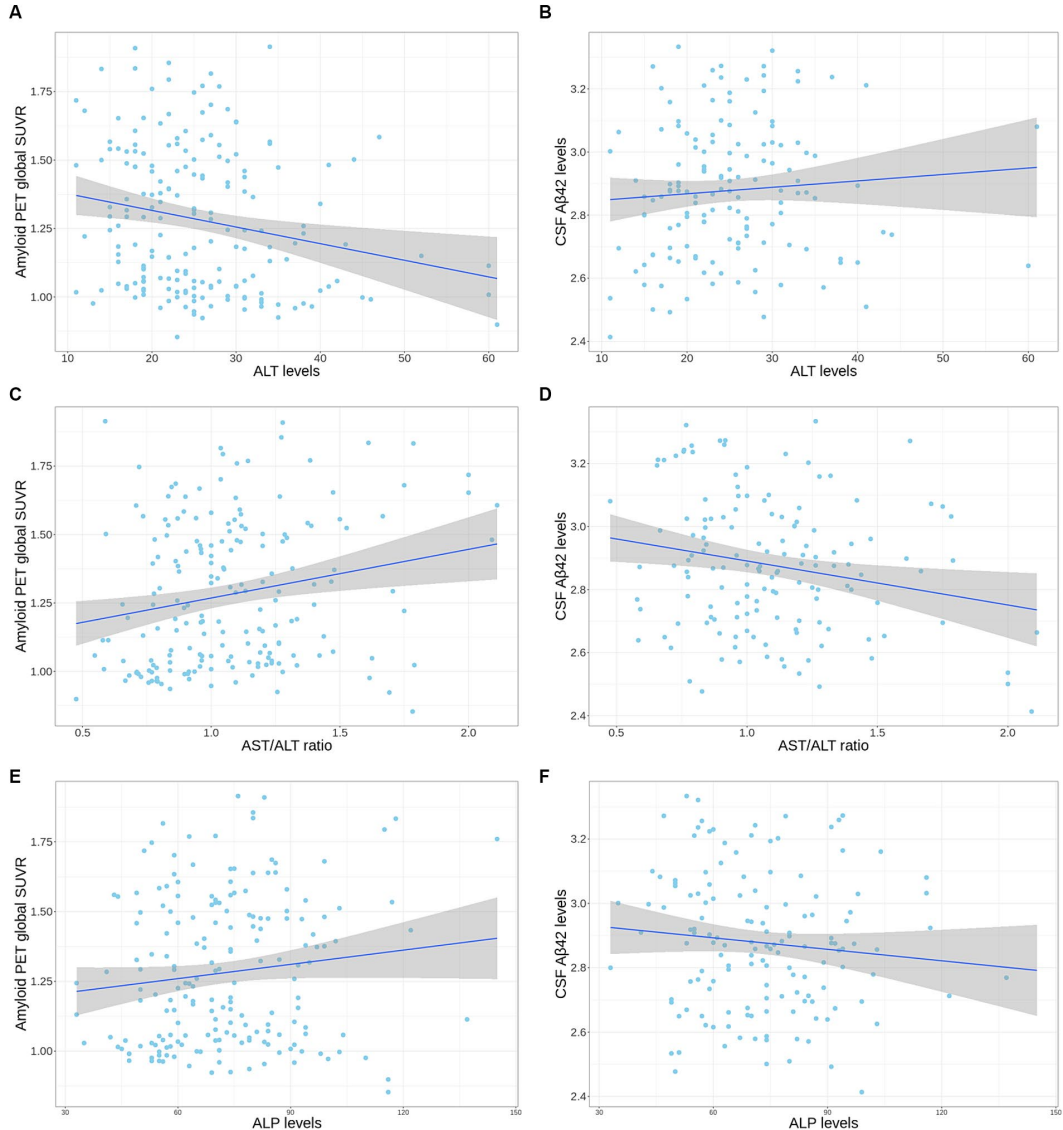
Correlation plot of liver enzymes levels with amyloid PET global SUVR and CSF Aβ42 levels in the *APOE* ε4 carrier group from the ADNI cohort. **(A,B)** The scatter plot represents a correlation of ALT levels with amyloid PET global SUVR **(A)** and CSF Aβ42 levels **(B)**. **(C,D)** The scatter plot represents a correlation of AST to ALT ratio with amyloid PET global SUVR **(C)** and CSF Aβ42 levels **(D)**. **(E,F)** The scatter plot represents a correlation of ALP levels with amyloid PET global SUVR **(E)** and CSF Aβ42 levels **(F)**. The gray zone around the linear regression line represents the 95% confidence interval. Aβ, amyloid-β; ADNI, Alzheimer’s Disease Neuroimaging Initiative; ALT, alanine aminotransferase; ALP, alkaline phosphatase; *APOE*, apolipoprotein E; CSF, cerebrospinal fluid; PET, positron emission tomography; SUVR, standard uptake value ratio.

In the ADNI cohort, lower ALT levels (*β* for linear regression coefficients [SE] = −0.006 [0.002], *p* = 0.02) and higher ALP levels (*β* [SE] = 0.002 [0.001], *p* = 0.046) and an AST to ALT ratio (*β* [SE] = 0.166 [0.061], *p* = 0.02) were significantly associated with higher values of amyloid PET global SUVR in the *APOE* ε4 carrier group after adjustment for multiple comparisons (median value: ALT, 23 U/L; ALP, 71 U/L; AST to ALT ratio, 1.04; IQR: ALT, 19–29 U/L; ALP, 59–85 U/L; AST to ALT ratio, 0.86–1.25) (Table 5 and Figure 1). Regarding diagnostic status, ALT levels were significantly decreased (estimate = −0.071, SE = 0.029, *p* = 0.03), while the AST to ALT ratio and ALP levels were significantly increased in AD compared with CN (AST to ALT ratio: estimate = 1.583, SE = 0.630, *p* = 0.03; ALP: estimate = 0.036, SE = 0.012, *p* = 0.02) in the *APOE* ε4 carrier group (Table 6). However, there were no significant associations between any of the six liver function markers and the diagnostic status or amyloid PET global SUVR in the *APOE* ε4 non-carrier group.

**Table 5:** Results of the association analysis of liver function markers with amyloid PET global SUVR in the ADNI cohort.

	Amyloid PET global SUVR			
	*APOE* ε4−^a^		*APOE* ε4+^a^	
	*β*^b^ (SE)	*P* ^c^	β^b^ (SE)	*P* ^c^
AST, U/L	0.002 (0.002)	0.82	−0.003 (0.003)	0.40
ALT, U/L	4.222 × 10^−4^ (0.001)	0.82	−0.006 (0.002)	0.02
AST to ALT ratio	0.026 (0.039)	0.82	0.166 (0.061)	0.02
ALP, U/L	−1.473 × 10^−4^ (0.001)	0.82	0.002 (0.001)	0.046
TB, mg/dL	−0.011 (0.050)	0.82	0.010 (0.079)	0.90
Albumin, g/dL	0.018 (0.041)	0.82	0.070 (0.072)	0.40

^a^After adjusting for age, sex, and body mass index.

^b^Linear regression coefficient of associations between liver function markers and amyloid PET global SUVR.

^c^Adjusted p value for FDR to correct for 6 tests of associations between liver enzymes and amyloid PET global SUVR.

ADNI, Alzheimer’s Disease Neuroimaging Initiative; ALP, alkaline phosphatase; ALT, alanine aminotransferase; APOE, apolipoprotein E; AST, aspartate aminotransferase; FDR, false discovery rate; PET, positron emission tomography; SE, standard error; SUVR, standard uptake value ratio; TB, total bilirubin.

**Table 6:** Results of the association analysis of liver function markers with AD diagnosis in the ADNI cohort.

	Diagnostic status (CN or AD)			
	*APOE* ε4−^a^		*APOE* ε4+^a^	
	Estimate^b^ (SE)	*P* ^c^	Estimate^b^ (SE)	*P* ^c^
AST, U/L	0.045 (0.044)	0.91	−0.014 (0.033)	0.67
ALT, U/L	0.007 (0.030)	0.95	−0.071 (0.029)	0.03
AST to ALT ratio	0.202 (0.932)	0.95	1.583 (0.630)	0.03
ALP, U/L	0.013 (0.13)	0.91	0.036 (0.012)	0.02
TB, mg/dL	−0.531 (1.369)	0.95	0.399 (0.806)	0.67
Albumin, g/dL	−0.058 (0.990)	0.95	−0.619 (0.845)	0.67

^a^After adjusting for age, sex, and body mass index.

^b^Logistic regression coefficients of association between liver function markers and diagnosis.

^c^Adjusted p value for FDR to correct for 6 tests of associations between liver enzymes and diagnosis.

AD, Alzheimer’s disease; ADNI, Alzheimer’s Disease Neuroimaging Initiative; ALP, alkaline phosphatase; ALT, alanine aminotransferase; APOE, apolipoprotein E; AST, aspartate aminotransferase; CN, cognitively normal; FDR, false discovery rate; SE, standard error; TB, total bilirubin.

### 3.3 Association analysis of liver enzymes with CSF biomarkers for AD

In the ADNI cohort, a high AST to ALT ratio was significantly associated with low CSF Aβ42 levels (*β* [SE] = −0.157 [0.055], *p* = 0.03) after adjustment for multiple comparisons (Table 7 and Figure 1). These associations were not observed with CSF p-tau_181_ and t-tau levels. Additionally, none of the six liver function markers showed significant associations with any CSF biomarkers for AD in the *APOE* ε4 non-carrier group.

**Table 7:** Results of the association analysis between liver function markers and CSF biomarkers for AD in the ADNI cohort.

	*APOE* ε4−^a^						*APOE* ε4+^a^					
	CSF Aβ42		CSF p-tau_181_		CSF t-tau		CSF Aβ42		CSF p-tau_181_		CSF t-tau	
	*β*^b^ (SE)	*P* ^c^	*β*^b^ (SE)	*P* ^c^	*β*^b^ (SE)	*P* ^c^	*β*^b^ (SE)	*P* ^c^	*β*^b^ (SE)	*P* ^c^	*β*^b^ (SE)	*P* ^c^
AST, U/L	0.002 (0.003)	0.92	−3.626 × 10^−4^ (0.002)	0.98	0.001 (0.002)	0.91	−0.003 (0.003)	0.43	−0.002 (0.003)	0.52	−0.002 (0.003)	0.41
ALT, U/L	0.002 (0.002)	0.92	−4.012 × 10^−4^ (0.001)	0.98	2.545 × 10^−4^ (0.001)	0.91	0.003 (0.002)	0.42	−0.003 (0.002)	0.46	−0.003 (0.002)	0.41
AST to ALT ratio	−0.038 (0.062)	0.92	0.030 (0.047)	0.98	0.031 (0.042)	0.91	−0.157 (0.055)	0.03	0.069 (0.057)	0.46	0.044 (0.048)	0.41
ALP, U/L	1.421 × 10^−5^ (0.001)	0.99	1.462 × 10^−4^ (0.001)	0.98	2.949 × 10^−4^ (0.001)	0.91	−0.002 (0.001)	0.22	0.001 (0.001)	0.46	0.001 (0.001)	0.41
TB, mg/dL	−0.001 (0.077)	0.99	0.048 (0.058)	0.98	0.042 (0.052)	0.91	−5.188 × 10^−4^ (0.077)	0.99	−0.056 (0.078)	0.52	−0.054 (0.066)	0.41
Albumin, g/dL	−0.030 (0.060)	0.92	−0.012 (0.046)	0.98	−0.005 (0.041)	0.91	−0.071 (0.070)	0.43	−0.046 (0.071)	0.52	−0.055 (0.060)	0.41

^a^After adjusting for age, sex, and body mass index.

^b^Linear regression coefficient of association between liver function markers and diagnosis.

^c^Adjusted *p* value for FDR to correct for 6 tests of associations between liver enzymes and each CSF biomarkers for AD.

Aβ, amyloid-β; AD, Alzheimer’s disease; ADNI, Alzheimer’s Disease Neuroimaging Initiative; ALT, alanine aminotransferase; ALP, alkaline phosphatase; APOE, apolipoprotein E; AST, aspartate aminotransferase; CSF, cerebrospinal fluid; FDR, false discovery rate; *p*-tau_181_, phosphorylated tau_181_; SE, standard error; t-tau, total tau; TB, total bilirubin.

### 3.4 Association analysis of liver enzymes with cognition

In the HUMC cohort, across all participants including those with SCD, MCI, and AD, low ALT levels were significantly associated with faster longitudinal decline in memory function (*β* [SE] = 0.035 [0.012], *p* = 0.029) in the *APOE* ε4 carrier group after adjustment for multiple comparisons (Table 8). Additionally, a high AST to ALT ratio was significantly associated with faster longitudinal decline in memory function (*β* [SE] = −1.012 [0.212], *p* < 0.001) in the *APOE* ε4 carrier group. There were no significant associations between cognitive performance and any of the six liver function markers in the *APOE* ε4 non-carrier group.

**Table 8:** Results of the association analysis of liver function markers with cognition in the HUMC cohort.

	Slope of cognitive performance							
	Language				Memory			
	*APOE* ε4−^a^		*APOE* ε4+^a^		*APOE* ε4−^a^		*APOE* ε4+^a^	
	*β*^b^ (SE)	*P* ^c^	*β*^b^ (SE)	*P* ^a^	*β*^b^ (SE)	*P* ^c^	*β*^b^ (SE)	*P* ^c^
AST, U/L	−0.005 (0.009)	0.763	0.009 (0.019)	0.679	0.001 (0.007)	0.887	−0.006 (0.011)	0.678
ALT, U/L	−0.003 (0.008)	0.862	0.029 (0.021)	0.414	−0.00 (0.006)	0.763	0.035 (0.012)	0.029
AST to ALT ratio	0.083 (0.134)	0.763	−0.412 (0.375)	0.439	0.100 (0.094)	0.763	−1.012 (0.212)	<0.001
ALP, U/L	−0.004 (0.003)	0.763	−4.439 × 10^−4^ (0.006)	0.940	−0.003 (0.002)	0.763	−0.003 (0.003)	0.539
TB, mg/dL	−0.043 (0.302)	0.887	0.715 (0.479)	0.414	0.155 (0.212)	0.763	−0.340 (0.283)	0.439
Albumin, g/dL	0.192 (0.217)	0.763	−0.384 (0.364)	0.439	0.158 (0.153)	0.763	−0.294 (0.215)	0.414

^a^After adjusting for age, sex, hypertension, diabetes mellitus, dyslipidemia, statin use, and education level.

^b^Coefficients of linear mixed-effects model of association between liver function markers and diagnosis across all participants including those with SCD, MCI, and AD.

^c^Adjusted *p* value for FDR for 12 tests of associations between liver enzymes and language and memory functions.

AD, Alzheimer’s disease; ALP, alkaline phosphatase; ALT, alanine aminotransferase; APOE, apolipoprotein E; AST, aspartate aminotransferase; FDR, false discovery rate; HUMC, Hallym University Medical Centers; MCI, mild cognitive impairment; SCD, subjective cognitive decline; SE, standard error.

In the ADNI cohort, among all participants, including those with CN, MCI, and AD, low ALT levels were significantly associated with faster longitudinal decline in memory function (*β* [SE] = 0.002 [0.001], *p* = 0.023) in the *APOE* ε4 carrier group after adjustment for multiple comparisons (Table 9). Moreover, a high AST to ALT ratio and ALP levels showed significant associations with faster longitudinal decline in language (AST to ALT ratio: *β* [SE] = −0.596 [0.236], *p* = 0.029; ALP: *β* [SE] = −0.011 [0.004], *p* = 0.023) and memory (AST to ALT ratio: *β* [SE] = −0.653 [0.224], *p* = 0.023; ALP: *β* [SE] = −0.001 [0.003], *p* = 0.023) functions in the *APOE* ε4 carrier group. However, there were no significant associations between cognitive performance and any of the six liver function markers in the *APOE* ε4 non-carrier group.

**Table 9:** Results of the association analysis of liver function markers with cognition in the ADNI cohort.

	Slope of cognitive performance							
	Language				Memory			
	*APOE* ε4−^a^		*APOE* ε4+^a^		*APOE* ε4−^a^		*APOE* ε4+^a^	
	*β*^b^ (SE)	*P* ^c^	*β*^b^ (SE)	*P* ^c^	*β*^b^ (SE)	*P* ^c^	*β*^b^ (SE)	*P* ^c^
AST, U/L	−0.011 (0.008)	0.474	0.004 (0.012)	0.836	−0.015 (0.008)	0.474	0.013 (0.012)	0.450
ALT, U/L	−0.004 (0.005)	0.703	0.016 (0.008)	0.118	−0.004 (0.005)	0.703	0.002 (0.001)	0.023
AST to ALT ratio	0.0004 (0.172)	0.998	−0.596 (0.236)	0.029	−0.094 (0.172)	0.703	−0.653 (0.224)	0.023
ALP, U/L	−0.002 (0.002)	0.703	−0.011 (0.004)	0.023	−0.004 (0.002)	0.474	−0.001 (0.003)	0.023
TB, mg/dL	0.376 (0.219)	0.474	−0.270 (0.302)	0.559	0.046 (0.220)	0.911	−0.009 (0.294)	0.977
Albumin, g/dL	0.172 (0.178)	0.703	0.120 (0.280)	0.802	0.098 (0.179)	0.703	0.194 (0.268)	0.625

^a^After adjusting for age, sex, body mass index, and education level.

^b^Coefficients of linear mixed-effects model of association between liver function markers and diagnosis among all participants, including those with CN, MCI, and AD.

^c^Adjusted *p* value for FDR for 12 tests of associations between liver enzymes and language and memory functions.

AD, Alzheimer’s disease; ADNI, Alzheimer’s Disease Neuroimaging Initiative; ALP, alkaline phosphatase; ALT, alanine aminotransferase; APOE, apolipoprotein E; AST, aspartate aminotransferase; CN, cognitively normal; FDR, false discovery rate; MCI, mild cognitive impairment; SE, standard error.

### 3.5 Mediation analysis of liver function markers on amyloid PET burden or AD diagnosis

**Figure 2:**
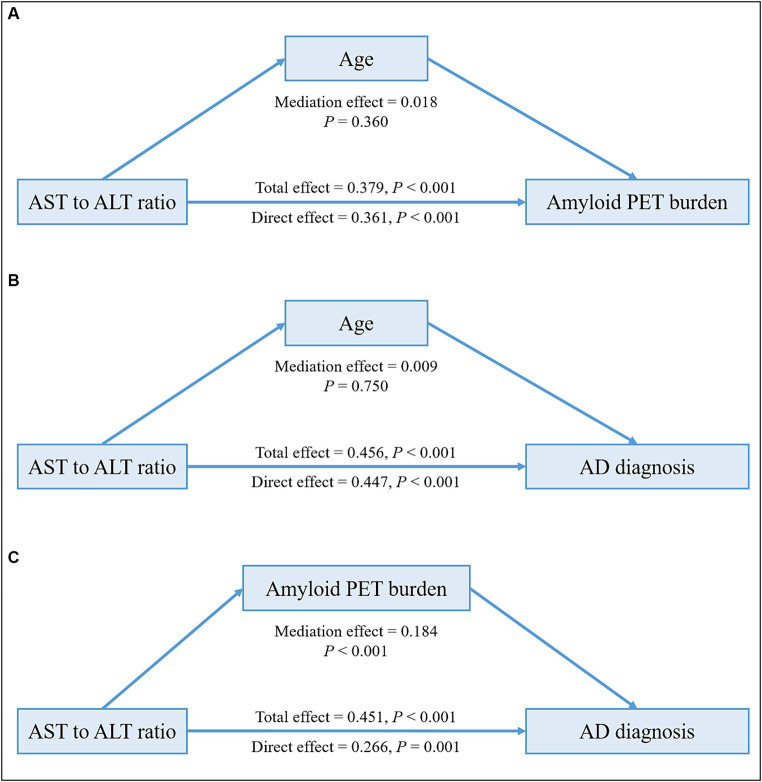
Mediation analysis of liver function markers on amyloid PET burden or diagnosis among the *APOE* ε4 carrier group in the HUMC cohort. Mediation effects of liver function markers on amyloid PET burden through age **(A)**, on AD diagnosis through age **(B)**, and on AD diagnosis through amyloid PET burden **(C)** in the *APOE* ε4 carrier group. All analyses were adjusted for age, sex, hypertension, diabetes mellitus, dyslipidemia, and statin use. AD, Alzheimer’s disease; ALT, alanine aminotransferase; *APOE*, apolipoprotein E; AST, aspartate aminotransferase; HUMC, Hallym University Medical Centers; PET, positron emission tomography.

In the HUMC cohort, mediation analyses revealed that the associations of ALT levels and the AST to ALT ratio with amyloid PET positivity (total effect: ALT; *β* = −0.009, *p* = 0.003; AST to ALT ratio; *β* = 0.379, *p* < 0.001; mediating effect: ALT; *β* = −0.001, *p* = 0.158; AST to ALT ratio; *β* = 0.018, *p* = 0.360) and AD diagnosis (total effect: ALT; *β* = −0.001, *p* < 0.001; AST to ALT ratio; *β* = 0.456, *p* < 0.001; mediating effect: ALT; *β* = −0.00004, *p* = 0.468; AST to ALT ratio; *β* = 0.009, *p* = 0.750) in the *APOE* ε4 carrier group were not mediated by age (Figure 2 and Table 10). Furthermore, the association between the AST to ALT ratio and AD diagnosis was significantly mediated by amyloid PET positivity (total effect: *β* = 0.451, *p* < 0.001; mediating effect: *β* = 0.184, *p* < 0.001), indicating a substantial indirect effect in the *APOE* ε4 carrier group.

**Table 10:** Results of the mediation analysis of liver function markers on amyloid PET positivity or diagnosis in the HUMC cohort.

			*APOE* ε4−^a^						*APOE* ε4+^a^					
			Mediation effect		Direct effect		Total effect		Mediation effect		Direct effect		Total effect	
Independent variable	Dependent variable	Mediator	Estimate	*P*	Estimate	*P*	Estimate	*P*	Estimate	*P*	Estimate	*P*	Estimate	*P*
ALT	Amyloid PET positivity	Age	0.00003	0.920	−0.003	0.220	−0.003	0.220	−0.001	0.158	−0.009	0.005	−0.009	0.003
AST to ALT ratio	Amyloid PET positivity	Age	0.002	0.042	0.048	0.079	0.050	0.076	0.018	0.360	0.361	<0.001	0.379	<0.001
ALT	AD diagnosis	Age	−0.001	0.046	−0.002	0.268	−0.003	0.114	−0.00004	0.468	−0.001	<0.001	−0.001	<0.001
AST to ALT ratio	AD diagnosis	Age	0.054	0.004	−0.019	0.729	0.035	0.576	0.009	0.750	0.447	<0.001	0.456	<0.001
ALT	AD diagnosis	Amyloid PET positivity	0.006	0.970	−0.002	0.290	0.004	0.820	−0.006	0.645	−0.001	0.059	−0.007	0.501
AST to ALT ratio	AD diagnosis	Amyloid PET positivity	0.008	0.550	−0.028	0.520	−0.020	0.680	0.184	<0.001	0.266	0.001	0.451	<0.001

^a^After adjusting for age, sex, hypertension, diabetes mellitus, dyslipidemia, and statin use.

^b^After adjusting for age, sex, hypertension, diabetes mellitus, dyslipidemia, statin use, and *APOE* ε4 carrier status.

AD, Alzheimer’s disease; ALT, alanine aminotransferase; APOE, apolipoprotein E; AST, aspartate aminotransferase; HUMC, Hallym University Medical Centers; PET, positron emission tomography.

**Figure 3:**
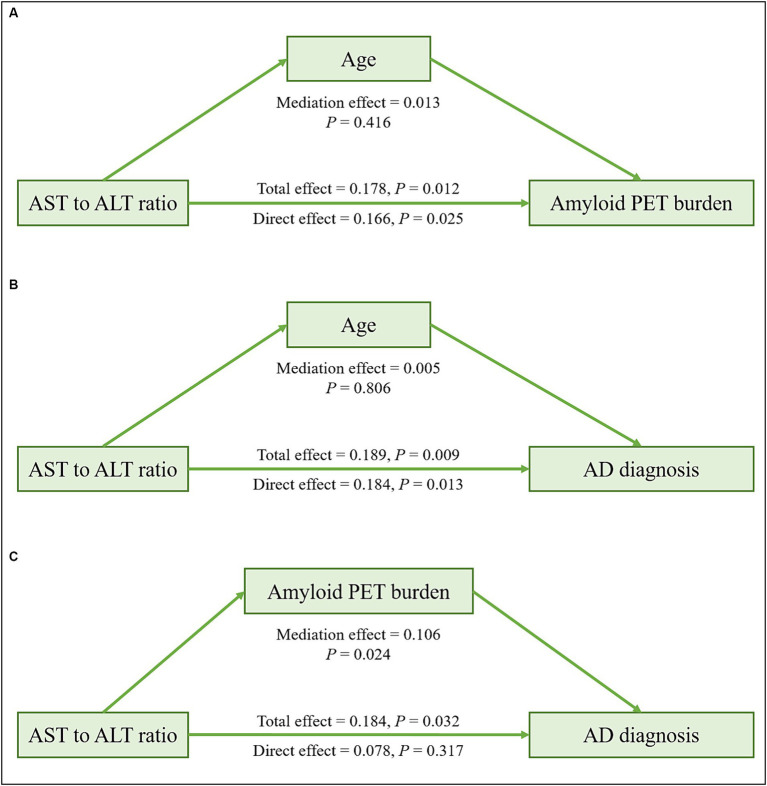
Mediation analysis of liver function markers on amyloid PET burden or diagnosis among the *APOE* ε4 carrier group in the ADNI cohort. Mediation effects of liver function markers on amyloid PET burden through age **(A)**, on AD diagnosis through age **(B)**, and on AD diagnosis through amyloid PET burden **(C)** in the *APOE* ε4 carrier group. All analyses were adjusted for age, sex, and body mass index. AD, Alzheimer’s disease; ADNI, Alzheimer’s Disease Neuroimaging Initiative; ALT, alanine aminotransferase; *APOE*, apolipoprotein E; AST, aspartate aminotransferase; PET, positron emission tomography.

In the ADNI cohort, mediation analyses revealed that the associations of ALT levels and the AST to ALT ratio with amyloid PET global SUVR (total effect: ALT; *β* = −0.006, *p* = 0.002; AST to ALT ratio; *β* = 0.178, *p* = 0.012; mediating effect: ALT; *β* = −0.0002, *p* = 0.464; AST to ALT ratio; *β* = 0.013, *p* = 0.416) and AD diagnosis (total effect: ALT; *β* = −0.013, *p* = 0.009; AST to ALT ratio; *β* = 0.189, *p* = 0.009; mediating effect: ALT; *β* = 0.0001, *p* = 0.922; AST to ALT ratio; *β* = 0.005, *p* = 0.806) were not mediated by age in the *APOE* ε4 carrier group (Figure 3 and Table 11). The associations of AD diagnosis with ALT (total effect: *β* = −0.013, *p* = 0.017; mediating effect: *β* = −0.007, *p* = 0.002) and the AST to ALT ratio (total effect: *β* = 0.184, *p* = 0.032; mediating effect: *β* = 0.106, *p* = 0.024) were significantly mediated by amyloid PET global SUVR, indicating a notable indirect effect in the *APOE* ε4 carrier group. Likewise, in the case of ALP levels, the association with AD diagnosis was not mediated by age but was significantly mediated by amyloid PET global SUVR (total effect: *β* = 0.0012, *p* = 0.002; mediating effect: *β* = 0.0004, *p* = 0.039), exclusively in the *APOE* ε4 carrier group (Table 11).

**Table 11:** Results of the mediation analysis of liver function markers on amyloid PET global SUVR or diagnosis in the ADNI cohort.

			*APOE* ε4−^a^						*APOE* ε4+^a^					
			Mediation effect		Direct effect		Total effect		Mediation effect		Direct effect		Total effect	
Independent variable	Dependent variable	Mediator	Estimate	*P*	Estimate	*P*	Estimate	*P*	Estimate	*P*	Estimate	*P*	Estimate	*P*
ALT	Amyloid PET global SUVR	Age	−0.001	0.003	0.0004	0.796	−0.0003	0.760	−0.0002	0.764	−0.006	0.002	−0.006	0.002
AST to ALT ratio	Amyloid PET global SUVR	Age	0.032	0.004	0.026	0.497	0.058	0.129	0.013	0.416	0.166	0.025	0.178	0.012
ALP	Amyloid PET global SUVR	Age	−0.0002	0.140	−0.0001	0.770	−0.0003	0.560	−0.0001	0.777	0.002	0.035	0.002	0.058
ALT	AD diagnosis	Age	−0.001	0.036	0.0002	0.934	−0.001	0.612	0.0001	0.922	−0.013	0.008	−0.013	0.009
AST to ALT ratio	AD diagnosis	Age	0.034	0.049	0.014	0.768	0.048	0.254	0.005	0.806	0.184	0.013	0.189	0.009
ALP	AD diagnosis	Age	−0.0001	0.230	0.0006	0.260	0.0005	0.390	−0.00002	0.871	0.001	0.003	0.001	0.003
ALT	AD diagnosis	Amyloid PET global SUVR	−0.0004	0.400	0.001	0.580	0.0006	0.730	−0.007	0.002	−0.006	0.229	−0.013	0.017
AST to ALT ratio	AD diagnosis	Amyloid PET global SUVR	0.020	0.220	−0.008	0.860	0.012	0.910	0.106	0.024	0.078	0.317	0.184	0.032
ALP	AD diagnosis	Amyloid PET global SUVR	0.0001	0.892	0.0003	0.075	0.0004	0.079	0.0004	0.039	0.0008	0.011	0.0012	0.002

^a^After adjusting for age, sex, and body mass index.

^b^After adjusting for age, sex, body mass index, and *APOE* ε4 carrier status.

AD, Alzheimer’s disease; ADNI, Alzheimer’s Disease Neuroimaging Initiative; ALP, alkaline phosphatase; ALT, alanine aminotransferase; APOE, apolipoprotein E; AST, aspartate aminotransferase; PET, positron emission tomography; SUVR, standard uptake value ratio.

## 4 Discussion

In this study, we comprehensively investigated the association of liver function markers with AD diagnosis, amyloid PET burden, CSF biomarkers for AD, and cognition in two independent cohorts. A particularly novel aspect of this study was its emphasis on how this association might differ based on the presence of the *APOE* ε4 allele.

The findings shed light on significant associations between liver enzyme levels, particularly ALT levels and the AST to ALT ratio, with AD diagnosis, amyloid PET burden, and cognitive function, especially among individuals carrying the *APOE* ε4 allele. However, these associations were not observed in those without the *APOE* ε4 allele. Furthermore, in the ADNI cohort, we observed that ALP levels were significantly associated with AD diagnosis, amyloid PET burden, and cognition, but this association was specific to the *APOE* ε4 carrier group. Additionally, the AST to ALT ratio was significantly associated with CSF Aβ42 levels in the ADNI cohort, especially in the *APOE* ε4 carrier group, but no such correlation was found for p-tau_181_ or t-tau levels. These findings strongly suggest that the presence of the *APOE* ε4 allele may play a crucial role in Aβ accumulation and cognitive decline in AD, potentially through its impact on liver function. The relationship between the *APOE* ε4 allele and liver enzymes might reflect underlying changes in hepatic lipid metabolism, which could subsequently influence cerebral Aβ aggregation and contribute to the neuropathology of AD (D’Alonzo et al., 2023).

Intriguingly, our mediation analyses, conducted in two independent cohorts, demonstrated that age did not mediate the associations of liver enzymes with amyloid PET burden and AD diagnosis in the *APOE* ε4 carrier group. Importantly, the associations between liver enzymes and AD diagnosis were partially mediated by the amyloid PET burden, and this mediation effect was particularly observed in the *APOE* ε4 carrier group.

This study has provided insights into the association of the *APOE* ε4 allele and serum liver enzymes with AD pathogenesis and longitudinal changes in cognition, as evidenced by the analysis of two independent cohorts. Moreover, our findings also highlighted that the effect of liver enzymes on AD was mediated through amyloid PET burden, with the impact of this mediation varying depending on whether an individual carries the *APOE* ε4 allele.

The *APOE* gene encodes APOE, a 35-kDa glycoprotein with widespread expression throughout the human body that serves as a key lipid transporter (Muñoz et al., 2019). Notably, the *APOE* ε4 allele is recognized as the most potent genetic risk factor for AD, and its influence increases in a gene dose-dependent manner (Farrer et al., 1997). In comparison with the *APOE* ε3 or ε2 allele, the *APOE* ε4 allele significantly increases the risk of AD by promoting the accumulation of Aβ in the brain (Castellano et al., 2011). Astrocytes are the primary source of APOE, which aids in the transportation of cholesterol to neurons via APOE receptors within the brain (Liu et al., 2013). Conversely, in peripheral tissues, hepatocytes are the primary producers of APOE, which is released into the bloodstream to regulate cholesterol metabolism in an isoform-dependent manner (Chernick et al., 2019). APOE, primarily generated by the liver, is distinct from the form found in the brain and separated by the blood–brain barrier (Chernick et al., 2019). Despite their physical separation, mounting evidence suggests that peripheral APOE could potentially influence insulin signaling, neuroinflammation, and synaptic function in the brain (Lane-Donovan et al., 2016; Giannisis et al., 2022). Moreover, plasma levels of APOE isoforms have been found to correlate with regional brain volume, cerebral glucose metabolism, and cognitive performance (Nielsen et al., 2017). In mice models, ApoE4 has been shown to impede the Aβ peripheral clearance (Sharman et al., 2010), while a biologically inspired nanostructure known as ApoE3-reconstituted high-density lipoprotein, which exhibits a strong binding affinity to Aβ, has been found to restore memory deficits by accelerating Aβ clearance (Song et al., 2014). Moreover, expression of ApoE4 in the liver has been found to exacerbate brain amyloid pathology, whereas liver-expressed ApoE3 has demonstrated beneficial effects on brain function and mitigated amyloid deposition in a mouse model (Liu et al., 2022). Despite these intriguing associations, there is no consensus regarding the exact relationship between the *APOE* allele and AD pathogenesis. For instance, Huynh et al. reported that the deletion of ApoE in the hepatocytes of APP/PS1 mice, resulting in decreased plasma ApoE levels but no change in brain ApoE levels, did not influence the amount of amyloid plaques (Huynh et al., 2019). Furthermore, there is supporting evidence that serum-based liver function markers, including AST, ALT, and ALP levels and the AST to ALT ratio, are associated with AD diagnosis, poor cognitive performance, and increased Aβ deposition (Kellett et al., 2011; Nho et al., 2019; Lu et al., 2021; Ferri et al., 2022; Han et al., 2022; Li et al., 2022). However, there is a lack of studies investigating the effects of the *APOE* ε4 allele on the association of liver function markers with AD pathogenesis and cognition in humans. Some of these concerns are partially addressed by the findings of our study, which showed that ALT levels and the AST to ALT ratio were significantly associated with AD diagnosis, Aβ accumulation, and cognition but only in the *APOE* ε4 carrier group across the two independent cohorts. Notably, such a correlation was not evident when assessing p-tau_181_ or t-tau levels. In particular, our mediation analysis revealed that the brain Aβ burden partially mediated the association between liver function markers and AD, exclusively in *APOE* ε4 carrier group. These findings indicate that liver function is associated with the accumulation of Aβ in the brain in AD, and this relationship depends on an individual’s *APOE* ε4 carrier status.

The primary endogenous peripheral receptor responsible for regulating plasma Aβ, thereby preventing Aβ access to the brain, is circulating low-density lipoprotein receptor (LDL)-related protein 1 (LRP1) (Tamaki et al., 2006; Zlokovic et al., 2010). In the liver, LRP1, in conjunction with LDL receptor, plays a crucial role in the clearance of circulating Aβ and APOE-containing particles from the bloodstream (Zlokovic et al., 2010; Van De Sluis et al., 2017). The decrease in LRP-1 expression is implicated in age-related decline in hepatic Aβ clearance (Tamaki et al., 2006). This impaired degradation of Aβ in the liver may lead to increased accumulation of Aβ in the brain (Maarouf et al., 2018). In our mediation analysis, we observed that the association of liver function markers with both brain Aβ burden and AD was not mediated by age, in the *APOE* ε4 carrier group. These results suggest that AD pathogenesis in *APOE* ε4 carriers is not predominantly driven by aging-related hepatic changes but rather through distinct mechanisms that impair hepatic Aβ clearance. As elucidated by D’Alonzo et al., the impact of the *APOE* ε4 allele on liver function involves impaired catabolism of lipoproteins, thereby increasing exposure to circulating lipoprotein-Aβ, which may lead to the Aβ aggregation in the brain and enhanced AD risk (D’Alonzo et al., 2023). Additionally, small high-density lipoproteins particles have been shown to influence brain Aβ levels, contributing to reduced AD risk through improved Aβ clearance and vascular function (Martinez et al., 2023).

The implications of liver function in the pathogenesis of AD offer intriguing insights into the potential therapeutic targets for AD. For instance, the notable therapeutic effects of the ayurvedic agent, *Withania somnifera*, achieved by increasing levels of liver LRP, suggests that targeting peripheral Aβ clearance may provide a unique approach to rapidly eliminate Aβ in AD transgenic mice (Sehgal et al., 2012). Statins have also demonstrated the potential to reduce the risk of AD by upregulating hepatic LRP1 and LDL receptor expression, which is mediated by sterol response element-binding protein-2 (Moon et al., 2011; Zissimopoulos et al., 2017). Additionally, transthyretin, a transporter protein primarily produced in the liver and released into the bloodstream, is downregulated in AD (Han et al., 2011). Given its role as a carrier of Aβ at the blood–brain barrier and in the liver, particularly via LRP1, transthyretin may offer valuable insights into the development of therapeutic strategies for AD (Alemi et al., 2016, 2017).

This study, while providing valuable insights into the relationship between *APOE* ε4 allele status, liver function tests, and AD biomarkers, is subject to several limitations that must be carefully considered. Firstly, the cross-sectional design of our analyses limits our ability to determine causality or temporal relationships among liver function, *APOE* ε4 allele status, and AD biomarkers. Moreover, while the HUMC and ADNI cohorts are typical of clinic participants, they may not represent the broader community, necessitating further validation of these results in more diverse socio-economic, educational, and racial groups. Secondly, our study was the inability to incorporate quantitative measures of amyloid PET in the HUMC cohort when investigating the relationship between liver enzymes and the amyloid PET burden. This limitation arises from the absence of imaging data, which restricts the depth of the analysis. However, the practice of visually rating amyloid PET scans remains valuable in the clinical setting. Thirdly, data on the presence of hepatitis were not available in both the HUMC and ADNI cohorts. Additionally, the ADNI cohort did not have data on presence of hypertension, diabetes mellitus, and dyslipidemia, while the HUMC cohort did not have data on BMI, which is an important covariate associated with ALT levels (Ndrepepa and Kastrati, 2019). Furthermore, the HUMC cohort did not have data on the CSF biomarkers for AD. Fourthly, the use of different cognitive tests to generate composite scores for distinct cognitive domains across cohorts, along with variations in diagnostic group frequencies and definitions, may have introduced variability and potential confounding factors into our results. Fifthly, the use of linear regression models in our study to investigate the association between liver enzyme levels and AD outcomes may not have adequately captured potential non-linear relationships. Lastly, our study did not fully address the potential role of gene–environment interactions, such as lifestyle factors like diet and alcohol use, in modulating the influence of *APOE* ε4 allele on AD risk and liver function, emphasizing the necessity for in-depth study on interventions to reduce AD risk.

## 5 Conclusion

In summary, our study across two independent cohorts provides valuable insights into the pivotal association of *APOE* ε4 status and liver enzymes with Aβ-related pathogenesis and cognition in AD. Future research should focus on unraveling the biological pathways at the intersection of liver function and AD, aiming to identify novel therapeutic targets that could mitigate the progression of AD.

## Data availability statement

The HUMC datasets used during the current study are not readily available due to their containing information that could compromise the privacy of research participants. Publicly available datasets from the ADNI cohort were analyzed in this study. This data can be found at: http://adni.loni.usc.edu.

## Ethics statement

The protocol for the HUMC study has been approved by the Clinical Research Ethics Committee of Chuncheon Sacred Heart Hospital, Hallym University (IRB No. 2023-07-009) and conforms to the provisions of the Declaration of Helsinki. All data sources mentioned in the ADNI studies are publically available summary level information that requires no ethical approval or consent. The ADNI obtained ethics approvals from all sites collecting data in accordance with the standards of the local ethics committee and Institutional Review Board boards.

## Author contributions

S-WH: Conceptualization, Data curation, Formal analysis, Investigation, Methodology, Writing – original draft, Writing – review & editing. S-HL: Writing – original draft, Writing – review & editing. JK: Writing – original draft, Writing – review & editing. J-JL: Writing – original draft, Writing – review & editing. YP: Writing – original draft, Writing – review & editing. SK: Writing – original draft, Writing – review & editing. KN: Writing – original draft, Writing – review & editing. J-HS: Conceptualization, Data curation, Formal analysis, Funding acquisition, Investigation, Methodology, Supervision, Writing – original draft, Writing – review & editing.

## Footnotes

^1^ http://adni.loni.usc.edu
